# Primary Pulmonary Hodgkin's Lymphoma Revealed by a Cavitary Lung Lesion: A Case With an Atypical Presentation and Literature Review

**DOI:** 10.7759/cureus.65479

**Published:** 2024-07-26

**Authors:** Boujemaa Razouq, Mouhsin Ibba, Mohamed Mahdaoui, Meryem El Ouazzani, Hicham Fenane, Yassine Msougar

**Affiliations:** 1 Thoracic Surgery, Mohammed VI University Hospital, Marrakesh, MAR; 2 Neurology, Mohammed VI University Hospital, Marrakesh, MAR; 3 Pathology, Mohammed VI University Hospital, Marrakesh, MAR

**Keywords:** hodgkin's lymphoma, pulmonary tumor, lymphoma, pulmonary hodgkin's lymphoma, cavitary pulmonary lesion

## Abstract

Primary pulmonary Hodgkin's lymphoma (PPHL) is an uncommon condition that accounts for less than 1% of all lymphomas. The clinical and radiological presentation of PPHL is nonspecific. This case report aimed to highlight the misleading presentation of PPHL, which initially manifested as a pulmonary cavitary lesion. The presented case report describes a perplexing presentation of PPHL in a 24-year-old female patient. Initially suspected to have necrotizing pneumonia or pulmonary tuberculosis due to symptoms including cough, hemoptysis, and weight loss, the patient underwent various diagnostic procedures, including bronchoscopy and CT-guided biopsy, which failed to provide a definitive diagnosis. Surgical resection was eventually pursued, leading to the conclusive identification of PPHL. This case highlights the diagnostic challenges associated with PPHL, emphasizing the importance of considering this rare entity in the differential diagnosis of pulmonary nodular or cavitary lesions. Prompt recognition and accurate diagnosis are essential for optimal management and improved patient outcomes. PPHL is an infrequent neoplasm that often presents diagnostic dilemmas. It requires consideration within the appropriate clinical framework to ensure timely diagnosis and intervention.

## Introduction

Hodgkin's lymphoma (HL) was initially described by Thomas Hodgkin in 1832 [1]. HL can be classified into classical (90%) and nodular lymphocyte-predominant types based on histological features and immunophenotypes [1]. Classical HL is characterized by the presence of Reed-Sternberg (RS) cells and comprises four subtypes: nodular sclerosis, lymphocyte rich, mixed cellularity, and lymphocyte depleted [1]. Lung involvement in HL is not uncommon, occurring in approximately 15-40% of cases [2]. Primary pulmonary Hodgkin's lymphoma (PPHL) occurs when lymphoid follicles or peribronchiolar adenopathies adjacent to the lung parenchyma extend into the lungs [3]. PPHL without peripheral lymphadenopathy or hepatosplenomegaly is an exceedingly rare disease, with only around 115 cases reported since 1927 [1]. In this case report, we present a perplexing and occult form of PPHL in a 24-year-old female patient, which presented a multitude of diagnostic challenges.

## Case presentation

**Figure 1:**
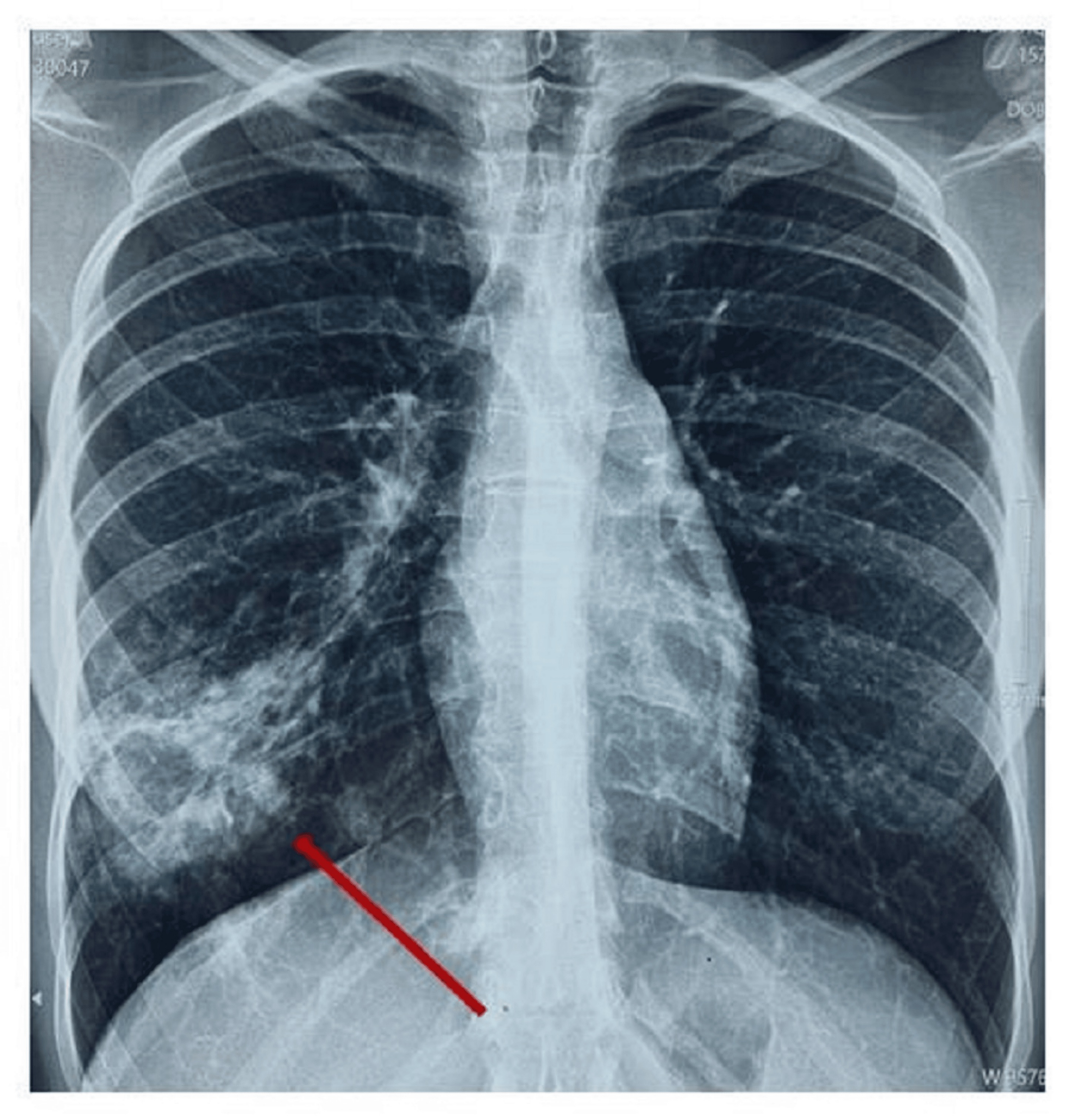
Chest X-ray image showing a cavitary lesion in the right lower lobe (red arrow).

A 24-year-old patient, previously treated for tuberculous lymphadenitis identified through a biopsy of the left supraclavicular adenopathy 11 years ago and declared cured, presented with worsening symptoms, including a productive cough with small episodes of hemoptysis, night sweats, and a weight loss of 5 kg over three months. There was no personal or family history of malignancy. Physical examination revealed decreased breath sounds in the right lower lung field with no palpable adenopathy or hepatosplenomegaly. A chest X-ray showed a cavitary lesion in the right lower lobe (Figure 1). 

**Figure 2:**
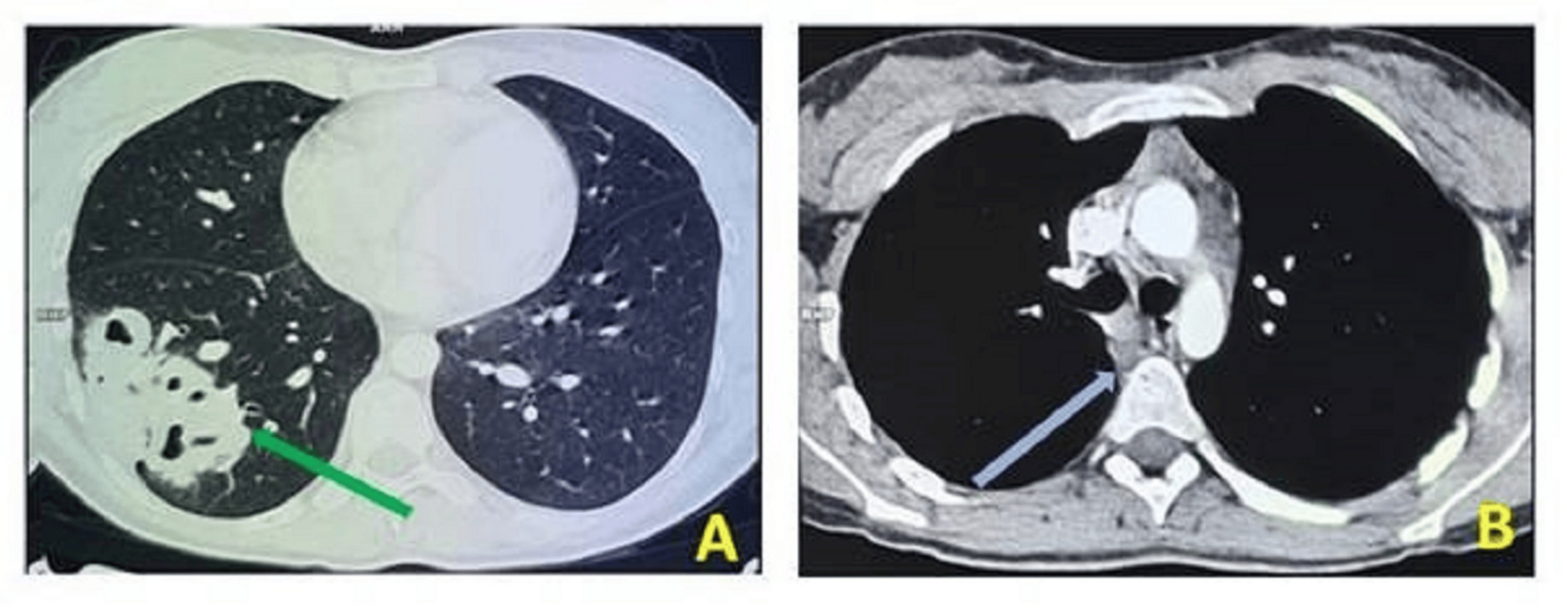
Thoracic CT image showing a heterogeneous mass with cavitation in the right lower lobe (green arrow), accompanied by Baréty's lodge adenopathies and subcarinal adenopathies (blue arrow).

Subsequently, a thoracic CT scan revealed a heterogeneous mass measuring 6.2×5.8×7.6 cm with cavitation in the right lower lobe, accompanied by Baréty's lodge adenopathies measuring 1.0 cm and subcarinal adenopathies measuring 1.3×1.0 cm (Figure 2).

Biological workup revealed hyperleukocytosis (14,140 mm/L) with a predominance of neutrophils (10,080 mm/L), a normal lymphocyte count of 1,824/L, and no anemia (Hb 12.2 g/dl). The platelet count was 300 thousand/L, the C-reactive protein level was 9.8 mg/L, the prothrombin level was 73%, and the lactate dehydrogenase level was within the normal range. Sputum *Mycobacterium tuberculosis* PCR and bacterial culture for *Mycobacterium tuberculosis* and human immunodeficiency virus (HIV) serology were negative. However, the serum QuantiFERON test was positive. Bronchoscopy with biopsy and bronchoalveolar lavage did not provide a definitive diagnosis.

Consequently, a CT-guided transparietal biopsy of the cavitary lesion was performed. *Mycobacterium tuberculosis* PCR, as well as bacteriological and mycological cultures, showed no signs of infection. Transparietal biopsy revealed extensive fibrosis in the interstitial lung parenchyma, as evidenced by the pathological findings. Some alveolar structures of varying sizes were still present, showing a typical lining of pneumocytic cells. The interstitium exhibited a dense and diffuse inflammatory infiltrate consisting of lymphocytes, histiocytes, and a significant number of eosinophilic polymorphs. Within the interstitium, granulomatous lesions with focal follicular organization were observed. These lesions primarily consisted of epithelioid cells mixed with debris from karyorrhectic nuclei, with rare atypical cells. The initial immunohistochemistry (IHC) results were inconclusive, suggesting a possible chronic inflammatory lesion, potentially of tubercular origin. The tumor showed low-level positivity for anti-CD3, anti-CD20, and anti-CD68 antibodies and 5% positivity for anti-Ki67. However, it did not express anti-pancytokeratin, anti-CD79, or anti-TTF1. Pulmonary tuberculosis was strongly suspected, and the patient was administered anti-bacillary drugs for three months. However, her symptoms persisted, and a subsequent CT scan confirmed the persistence of the disease, with an increase in the size of the lesion to 6.7×6.3×8.1 cm. Right laterotracheal adenopathies measuring 1.1 cm, Baréty's lodge measuring 0.9 cm, and subcarinal adenopathies measuring 1.3×2.0 cm were also identified. Abdominopelvic and brain scans were unremarkable.

Surgery was performed based on a strong suspicion of necrotizing pneumonia with hemoptysis, the lack of a definitive microbiological diagnosis, a negative CT-guided transparietal lung biopsy result, and the failure of medical interventions. However, video-assisted thoracoscopy was impeded by the presence of dense pleural adhesions. Instead, a posterolateral thoracotomy was conducted by accessing the sixth intercostal space, revealing hepatization of the right lower lobe with a firm, indistinct mass measuring 6.0 cm. Subsequently, a right lower lobectomy was performed after pneumolysis. Postoperative progression was uncomplicated, with the removal of the chest drain and the patient's discharge occurring on the fifth day following the surgical procedure.

The gross examination revealed a right lower lobectomy weighing 400 grams and measuring 11.0×9.0×6.0 cm. Upon sectioning, a poorly defined, whitish, nodular lesion measuring 6.0×6.0×5.8 cm was identified. The lesion exhibited firm consistency and displayed signs of hemorrhagic and necrotic changes. It was located 0.5 cm from the surgical margin and 0.2 cm from the visceral pleura.

Microscopic examination revealed that the lung parenchyma served as the site for malignant proliferation within a heterogeneous granulomatous background. This background consisted of lymphocytes, plasma cells, histiocytes, sporadic neutrophils, and eosinophils, accompanied by a significant number of enlarged tumor cells. These tumor cells exhibited characteristics of binucleated RS cells as well as lacunar Hodgkin cells.

Immunohistochemical studies revealed the absence of anti-cytokeratin and anti-CD20 antibodies in the tumor cells. However, aberrant expression of the anti-CD3 antibody was noted in the tumor lymphoid cells. Additionally, there was a strong cytoplasmic expression of anti-CD30 and anti-CD15 antibodies with perinuclear enhancement. Moderate membranous expression of anti-CD68 was observed in the histiocytic cells, while moderate focal nuclear expression of anti-PAX5 was observed. There was no expression of anti-CD246. Morphological appearance suggests classical HL, and the cells of interest expressing aberrant CD3 do not present anaplasia.

**Figure 3:**
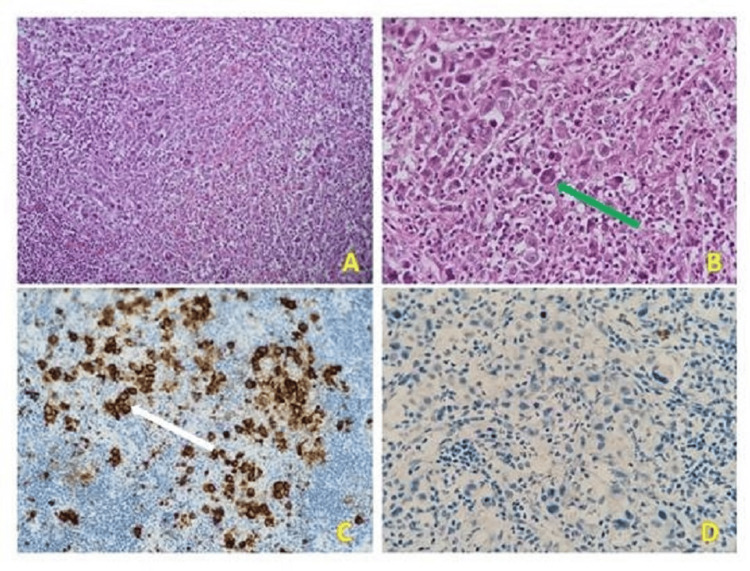
(A) Granulomatous lesions with focal follicular organization in the interstitium (H&E stain, 100× magnifications). (B) Large cell tumor proliferation displaying characteristic binucleated Reed-Sternberg cells (H&E stain, 400× magnifications) (green arrow). (C) Cytoplasmic expression with perinuclear enhancement of CD30 by tumor cells (IHC, 400× magnifications) (white arrow). (D) Absence of cytokeratin in tumor cells (IHC, 400× magnifications). IHC: immunohistochemistry

In conclusion, this case represents a pulmonary localization of classical HL with mixed cellularity and aberrant expression of the T-cell marker, according to the latest World Health Organization classification (WHO 2022) (Figure 3).

**Figure 4:**
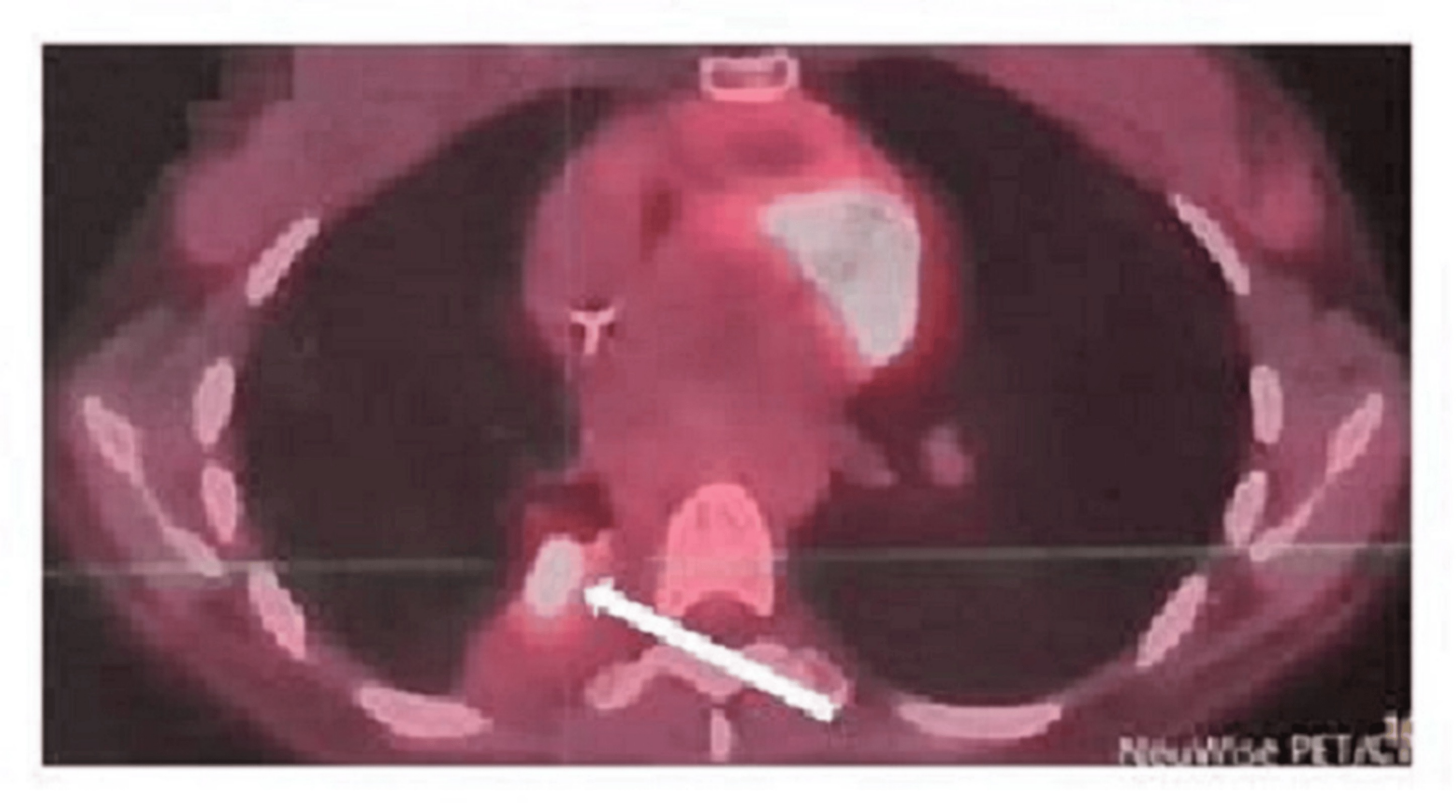
PET/CT scan before chemotherapy indicating hypermetabolic pleuropulmonary lymphomatous dissemination (white arrow).

One month later, a PET/CT fluorodeoxyglucose (FDG) scan indicated lymphomatous dissemination at several sites. These included a hypermetabolic pleuropulmonary lesion (Figure 4) and hypermetabolic adenopathies in the upper right paratracheal and subcarinal and the right lower jugular and supraclavicular region, measuring 1.2 cm in size with a maximum standardized uptake value (SUVmax) of 17.8. Additionally, a right internal iliac pathological hypermetabolic adenopathy with dimensions of 1.1×2.0 cm was observed, with an SUV max of 13.6.

**Figure 5:**
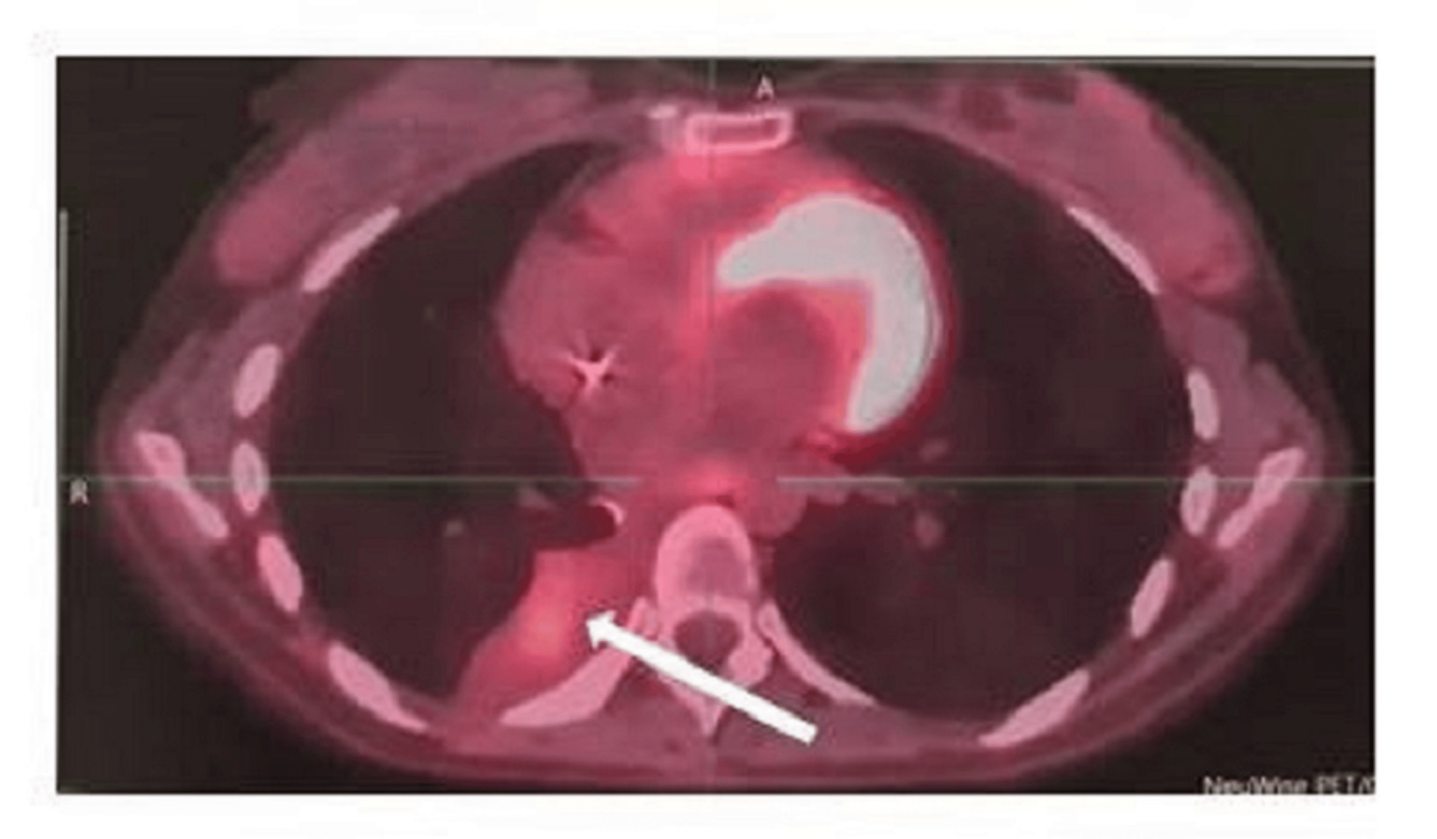
PET/CT scan after the end of treatment revealing a favorable metabolic response and regression of pulmonary metabolic activity (white arrow).

Epstein-Barr virus (EBV) serology was negative. Based on these findings, a diagnosis of PPHL was made (stage IV classical HL), and the patient was treated with two cycles of adriamycin, bleomycin, vinblastine, and dacarbazine (ABVD); due to the unavailability of brentuximab vedotin, we eliminated the bleomycin in the last two cycles. Interim PET was obtained and indicated that FDG uptake is less than the mediastinal blood pool (Deauville score of 2). Furthermore, a follow-up PET/CT scan after the end of treatment revealed a favorable metabolic response (Deauville score of 2), showing regression of pulmonary metabolic activity and clear morpho-metabolic regression of mediastinal lymph node involvement (Figure 5). No new suspicious hypermetabolic foci were identified. Follow-up radiographs at one, three, and six months demonstrated the disappearance of the right basal opacity.

## Discussion

PPHL is characterized by the clonal proliferation of lymphoid cells in the lung parenchyma without metastasis to other sites at the time of diagnosis [4]. It represents a rare subset of primary lung malignancies, accounting for less than 0.5% of all cases and comprising less than 1% of pulmonary lymphomas [2]. Due to the limited number of reported cases, the clinical features of PPHL are not well defined [1]. The diagnosis of PPHL requires the fulfillment of three criteria: (1) histological features consistent with HL involving the lung parenchyma, (2) limited disease confined to the lungs with minimal lymph node involvement, and (3) absence of disease in other organs [4]. In our patient, two criteria were met, and lymph node involvement was moderate which is atypical for PPHL.

Analysis of 115 patients revealed a greater incidence of PPHL among women than among men, with 64 cases (55.7%) occurring in females and 51 cases (44.3%) occurring in males, resulting in a female-to-male ratio of approximately 1.25:1. Notably, the age distribution showed a peak in the 20s. Among the examined patients, nodular sclerosis was identified as the predominant subtype, accounting for 37.4% (43 patients) of the total, followed by mixed cellularity at 17.4% (20 patients) [1]. However, our patient presented with PPHL of the mixed cellularity subtype.

Typical symptoms associated with PPHL include cough, dyspnea, weight loss, fever, night sweats, and, frequently, hemoptysis [2,5]. Additionally, patients may present with either asymptomatic or nonspecific systemic and respiratory symptoms. [6]. In our case, the patient presented with hemoptysis, weight loss, fever, and night sweats, closely mimicking the clinical presentation commonly associated with pulmonary tuberculosis.

The clinical and radiological presentation of PPHL can sometimes resemble infectious etiologies, such as pulmonary tuberculosis and inflammation, potentially complicating the diagnostic process [7]. Our case shares similarities with pulmonary tuberculosis. PPHL can also mimic other conditions, such as pneumonia, lung carcinoma, or metastases, presenting as one or multiple nodules or, in rare cases, as excavated lesions, often observed during chemotherapy due to necrosis. Notably, the initial presentation of our patient with a cavitary lung lesion represents an unusual manifestation of PPHL [6]. PPHL typically affects the upper regions of the lung. Conversely, secondary lung involvement of the HL manifests as a more dispersed miliary distribution, lacking a specific preference for any particular zone [8]. Nevertheless, in the present case, the localization in the lower lobe was considered atypical.

PPHL should be considered a potential diagnosis in patients who present with pulmonary nodular or cavitary lesions without a microbiological diagnosis and who are unresponsive to anti-bacillary drugs, particularly in the absence of known tuberculosis contact or family history [4]. In this particular patient, the presence of a previous history of lymph node tuberculosis increased the possibility of recurrence; however, the lack of improvement with anti-bacillary drugs justifies the discontinuation of treatment and the need for further investigations to ascertain the underlying cause.

The diagnosis of PPHL is often delayed due to the nonspecific nature of radiological examinations and bronchoscopy findings. In the largest series conducted by Radin et al., only one out of 39 bronchoscopies yielded a conclusive diagnostic outcome [6]. However, pathologists encounter ongoing challenges in diagnosing this intricate disease due to the constraints imposed by limited samples. The identification of Hodgkin or RS cells in small biopsies or intraoperative frozen sections presents a significant challenge [1].

In our patient, surgery was pursued due to a strong suspicion of necrotizing pneumonia with hemoptysis, compounded by the absence of a definitive microbiological diagnosis despite a negative CT-guided transparietal lung biopsy result and unsuccessful medical interventions. After surgical resection, the diagnosis was conclusively established. This experience underscores the significance of resorting to an open lung biopsy or surgical resection for suspicious lesions to attain a definitive diagnosis, especially when the lymph node is inaccessible for a needle biopsy or if an initial biopsy is not adequate, as advocated by the current literature [5].

IHC plays a pivotal role in establishing a definitive diagnosis in patients who present distinct histopathological features. Regarding PPHL, neoplastic cells exhibit positivity for CD15, CD30, Pax5, and occasionally CD20 but concurrently lack expression of T-cell markers [8]. However, our patient exhibited the presence of anti-CD30 and anti-CD15 antibodies associated with the aberrant expression of anti-CD3 which is associated with decreased event-free survival and overall survival [9].

Previous studies have reported EBV infection in 30-50% of all HL patients, although the exact role of EBV in the pathogenesis of HL remains uncertain [8]. In our patient, EBV serology returned negative results.

Management guidelines for PPHL have been established, and patients are generally managed according to these guidelines. Several prognostic factors associated with poorer outcomes in patients with PPHL, including older age, bilateral involvement, the presence of multiple lesions, pleural effusion, and cavitation, have been identified [2].

## Conclusions

PPHL is a rare manifestation of HL that poses diagnostic and therapeutic challenges. This case report illustrates the complexity of diagnosing PPHL, particularly when mimicking other pulmonary diseases such as tuberculosis. Prompt recognition and accurate diagnosis are essential for appropriate management and improved patient outcomes. Awareness of PPHL among clinicians and pathologists is crucial to avoid delays in diagnosis and ensure timely initiation of treatment. Further research is warranted to elucidate the underlying pathogenesis and optimize therapeutic approaches for this rare entity.

## References

[1] Jung H, Kim HS, Han J, Ko YH, Choi YD, Lee T (2022). Clinicopathological characteristics of primary pulmonary Hodgkin lymphoma (PPHL): two institutional experiences with comprehensive literature review of 115 PPHL cases. J Clin Med.

[2] Tanveer S, El Damati A, El Baz A, Alsayyah A, ElSharkawy T, Regal M (2015). Primary pulmonary Hodgkin lymphoma. Rare Tumors.

[3] Lluch-Garcia R, Briones-Gomez A, Castellano EM, Sanchez-Toril F, Lopez A, Brotons B (2010). Primary pulmonary Hodgkin's lymphoma. Can Respir J.

[4] Chiu WC, Chen SH, Chen BJ, Huang YL, Miserc JS, Wei CH, Lin WC (2021). Primary pulmonary Hodgkin's lymphoma: a rare etiology mimicking pulmonary tuberculosis. Pediatr Neonatol.

[5] Sławiński L, Sołek JM, Miłkowska-Dymanowska J, Jesionek-Kupnicka D, Góra-Tybor J, Mikulski D, Braun M (2023). Primary pulmonary Hodgkin's lymphoma mimicking granulomatosis with polyangiitis - a case report of diagnostic and therapeutic dilemmas. Contemp Oncol (Pozn).

[6] El Hage H, Hossri S, Samra B, El-Sayegh D (2017). Primary pulmonary Hodgkin's lymphoma: a rare etiology of a cavitary lung mass. Cureus.

[7] Jain E, Al-Tarbsheh AH, Oweis J, Jacobson E, Shkolnik B (2021). Hodgkin's lymphoma presenting as multiple cavitary lung lesions. Eur J Case Rep Intern Med.

[8] Cooksley N, Judge DJ, Brown J (2014). Primary pulmonary Hodgkin's lymphoma and a review of the literature since 2006. BMJ Case Rep.

[9] Venkataraman G, Song JY, Tzankov A (2013). Aberrant T-cell antigen expression in classical Hodgkin lymphoma is associated with decreased event-free survival and overall survival. Blood.

